# Incremental prognostic value of echocardiography of left ventricular remodeling and diastolic function in STICH trial

**DOI:** 10.1186/s12947-020-00195-1

**Published:** 2020-05-28

**Authors:** Kyung-Hee Kim, Lilin She, Kerry L. Lee, Rafal Dabrowski, Paul A. Grayburn, Miroslaw Rajda, David L. Prior, Patrice Desvigne-Nickens, William A. Zoghbi, Michele Senni, Guglielmo Stefanelli, Cesare Beghi, Thao Huynh, Eric J. Velazquez, Jae K. Oh, Grace Lin

**Affiliations:** 1 Division of Cardiovascular Diseases, Sejong General Hospital, Bucheon, South Korea; 2 Division of Cardiovascular Diseases, Echocardiography Core Laboratory, Mayo Clinic, Gonda 6 South, 200 First St SW, Rochester, MN USA; 3 Duke Clinical Research Institute, Duke University Medical Center, Durham, NC USA; 4 National Institute of Cardiology, Warsaw, Poland; 5 Baylor University Medical Center, Dallas, TX USA; 6 Capital Health Queen Elizabeth II Health Sciences Centre, Halifax, Canada; 7 St. Vincent’s Hospital, Melbourne, Australia; 8 Washington, DC USA; 9 Cardiovascular Imaging Institute, Houston Methodist DeBakey Heart and Vascular Center, Houston, TX USA; 10 Hospital, Papa Giovanni XXIII, Bergamo, Italy; 11 Hesperia Hospital, Modena, Italy; 12 Ospedale di Circolo, University of Insubria, Varese, Italy; 13 Montreal General Hospital, McGill Health University Center, Montreal, Canada; 14 Yale School of Medicine, New Haven, CT USA

**Keywords:** Heart failure, Diastolic dysfunction., Ischemic cardiomyopathy

## Abstract

**Aims:**

We sought to determine which echocardiographic markers of left ventricular (LV) remodeling and diastolic dysfunction can contribute as incremental and independent prognostic information in addition to current clinical risk markers of ischemic LV systolic dysfunction in the Surgical Treatment for Ischemic Heart Failure (STICH) trial.

**Methods and results:**

The cohort consisted of 1511 of 2136 patients in STICH for whom baseline transmitral Doppler (E/A ratio) could be measured by an echocardiographic core laboratory blinded to treatment and outcomes, and prognostic value of echocardiographic variables was determined by a Cox regression model. E/A ratio was the most significant predictor of mortality amongst diastolic variables with lowest mortality for E/A closest 0.8, although mortality was consistently low for E/A 0.6 to 1.0. Mortality increased for E/A < 0.6 and > 1.0 up to approximately 2.3, beyond which there was no further increase in risk. Larger LV end-systolic volume index (LVESVI) and E/A < 0.6 and > 1.0 had incremental negative effects on mortality when added to a clinical multivariable model, where creatinine, LVESVI, age, and E/A ratio accounted for 74% of the prognostic information for predicting risk. LVESVI and E/A ratio were stronger predictors of prognosis than New York Heart Association functional class, anemia, diabetes, history of atrial fibrillation, and stroke.

**Conclusions:**

Echocardiographic markers of advanced LV remodeling and diastolic dysfunction added incremental prognostic value to current clinical risk markers. LVESVI and E/A ratio outperformed other markers and should be considered as standard in assessing risks in ischemic heart failure. E/A closest to 0.8 was the most optimal filling pattern.

## Highlights

The Surgical Treatment for Ischemic Heart Failure (STICH) trial represents one of the largest cohorts of ischemic cardiomyopathy.

Diastolic dysfunction of advanced LV remodeling can contribute incremental prognostic value to current clinical markers of heart failure severity: these may have different effects on patients treated with CABG vs medical therapy, but does not impact outcomes differently in patients treated with CABG alone vs CABG+ SVR.

Inclusion of E/A ratio and LVESVI could enhance prognostic models for ischemic heart failure.

E/A ratio closest to 0.8 is the most optimal filling pattern in ischemic HFrEF.

## Introduction

Prognosis in heart failure with reduced ejection fraction (HFrEF) due to ischemic cardiomyopathy is affected by the severity of left ventricular (LV) remodeling as well as clinical co-morbidities including anemia and renal failure [1, 2]. Although many echocardiographic markers of LV remodeling, including LV size and geometry, functional mitral regurgitation (MR), diastolic dysfunction, and right ventricular (RV) dysfunction, are known to impact on mortality in HFrEF [3–6], only EF is included in most clinical HFrEF prognostic models [7, 8]. Whether the inclusion of diastolic filling parameters or other echocardiographic variables added to current clinical risk markers will have incremental prognostic value is not well defined. The Surgical Treatment for Ischemic Heart Failure (STICH) trial represents one of the largest cohorts of patients with HFrEF due to ischemic cardiomyopathy and is therefore an ideal population in which to determine the incremental prognostic value of echocardiographic markers of LV remodeling when combined with clinical risk markers. We hypothesized that diastolic filling parameters would remain the most prognostically significant marker amongst other markers in ischemic HFrEF.

## Methods

### Patient selection

The STICH study design has been described [9, 10]. Between July 2002 and May 2007, 2136 patients with ischemic HFrEF (LVEF ≤35%) amenable to coronary artery bypass grafting (CABG) were enrolled from 127 clinical centers in 26 countries. Patients were stratified into the Hypothesis 1 (H1; CABG vs. medical therapy) and Hypothesis 2 (H2; CABG vs. CABG + surgical ventricular reconstruction) cohorts. Eligibility for surgical ventricular reconstruction (SVR) was determined by the presence of dominant anterior/ apical dyskinesia; on this basis 1000 patients were enrolled into H2 [9, 11]. Only the 1511 patients from both cohorts with adequate baseline echocardiographic systolic, diastolic left ventricular (LV) function and RV function assessment within 90 days of randomization were included.

### Echocardiography Core lab analysis

Echocardiographic data for STICH was prospectively acquired using a standardized protocol and a comprehensive list of baseline measurements including the number of patients feasible measurements was reported [4]. MR and RV systolic function were graded qualitatively; quantitative measurements were not prospectively acquired [3]. RV function was assessed prospectively by visual interpretation and categorized as normal, mild, moderate, or severe dysfunction. The appreciation of the overall mechanical function of the RV was mainly based on the extent of RV free wall segmental motion, wall thickening, RV cavity size, and subjective assessment of RV area change (normal> 50%, mild 30–50%, moderate 20–30%, and severe < 20% from diastole to systole). RV assessment was derived from the parasternal long-axis, apical 4-chamber, and subcostal views. This assessment was based on visual assessment by an experienced Echocardiography Core Laboratory physician [12, 13]. The severity of MR was primarily determined by the physician’s visual assessment of width, depth, and area of mitral regurgitation jet. In addition, effective regurgitant orifice (ERO) was determined using the PISA (proximal isovelocity surface area) method, as previously described whenever possible.

The Mayo Clinic Echocardiography Core Laboratory (Rochester, MN) analyzed echocardiographic data in a blinded fashion without knowledge of clinical or laboratory data and quality assurance methods have been described [3, 14, 15]. Patients in atrial fibrillation or who had undergone previous mitral valve surgery were excluded from diastolic function analysis.

Eleven echocardiographic variables were selected for analysis based on prior data: E and A velocity, E/A ratio, deceleration time, mitral annular e’ velocity, E/e’ ratio, LVEF, LV end-systolic volume index (LVESVI), LV end-diastolic volume index (LVEDVI), sphericity index, RV systolic function, severity of MR, and left atrial volume index (area-length method) [3–6, 16–18]. Estimated RV systolic pressure was measurable in only 449 of 2136 patients and was excluded from analysis [3].

### Statistical analysis

Data were summarized using the mean and standard deviation or the median with 25th and 75th percentiles for continuous variables and frequencies and percentages for categorical variables. The distribution of continuous variables amongst groups was compared by the non-parametric Kruskal-Wallis test, and categorical variables with conventional chi-square statistics.

The relationship of each variable to mortality was assessed with the Cox regression model [19]. Linearity or non-linearity of the relationship with respect to the log hazard ratio was assessed using restricted cubic spline functions within the framework of the Cox model [20]. The strength of these relationships was characterized using chi-square statistics obtained from the modeling process. Both univariable and multivariable modeling were performed. Relative risks were expressed as hazard ratios with associated 95% confidence intervals and were generated from the Cox model. Due to missing data from suboptimal echo images 25 datasets were created and combined using multiple imputation and analyses performed for both the original and imputed datasets [21].

The multivariable model included clinical prognostic markers: age, creatinine, hemoglobin, history of stroke, history of atrial fibrillation, diabetes, NYHA functional class, and treatment (medical vs. surgical therapy, with surgical therapy including CABG or CABG + SVR), as well as the STICH risk at randomization (RAR) index [1, 2, 7, 10, 11, 22, 23]. All analyses were performed using SAS statistical software, version 9.4 (SAS Institute Inc., Cary, NC).

## Results

### Study population

We reported baseline demographics and clinical data of the 2136 patients in STICH [3, 11, 23]. Baseline echocardiography was performed within 90 days of randomization and prior to initiating study treatment in 2009 (94% of 2136) patients, of which 498 (24.7% of 2009) were excluded due to technically inadequate E/A ratio measurement or absent A wave due to atrial fibrillation, the remaining 1511 patients comprised our cohort. The primary outcome was the rate of death from any cause. There were 604 deaths over a median follow-up of 56 months. Median age was 60 years (25th percentile; 54, 75th percentile; 68) and 84.6% were male. The median E/A ratio was 1.0 (25th percentile; 0.67, 75th percentile; 1.67) with no difference between the H1 and H2 populations with respect to the distribution of this variable [3]. At least moderate MR (≥grade 2) was present in 24.1% (344 of 1426 patients in whom MR was characterized) in the cohort, at least moderate MR was also present in 29.4% (*p* = 0.033 between groups) in the 498 patients excluded due to absent E/A ratio (118 of 402).

Median values for the eleven echocardiographic variables are shown (Table 1). By study design, E/A ratio was the most complete dataset, whereas E/e’ ratio had the most missing values.

**Table 1:** Baseline Echo Variables^a^

Echo Variables	N	Data Distribution
E/A ratio	1511	1.00 (0.67, 1.67)
E velocity (m/s)	1511	0.70 (0.50, 0.90)
EF (%)	1498	29.0 (24.0, 35.0)
MR severity (by CFI grade)	1426	
Grade 0		404 (28.3%)
Grade 1		678 (47.6%)
Grade 2		233 (16.3%)
Grade 3		78 (5.5%)
Grade 4		33 (2.3%)
RV dysfunction	1414	
Normal		1069 (77.5%)
Mild dysfunction		181 (12.8%)
Moderate dysfunction		102 (7.2%)
Severe dysfunction		35 (2.5%)
Deceleration time (ms)	1386	181.0 (149.0, 221.0)
LVEDVI (cc/m^2^)	1161	111.8 (90.5, 134.0)
LVESVI (cc/m^2^)	1161	79.1 (60.8, 99.4)
LA volume index (cc/m^2^)	947	39.3 (30.8, 48.6)
Sphericity index	935	0.7 (0.6, 0.7)
E/e’ ratio	873	15.0 (11.2, 20.0)

^a^Continuous variables are presented as Median (25th, 75th percentile), and categorical variables are presented as N and %

*EF* Ejection fraction, *MR* Mitral regurgitation, *RV* Right ventricule, *LVEDVI* Left ventricular end diastolic volume index, *LVESVI* Left ventricular end systolic volume index, *LA* left atrium

### Univariable modeling of prognostic variables with the original dataset

Each of the eleven variables, when considered without imputation, was associated with all- cause mortality. LVESVI had a strong relationship with mortality (χ^2^ = 57.5, *p* < 0.001) even with a large number of missing values (350 patients), as did EF (χ^2^ = 43.5, *p* < 0.001) and E/A ratio (χ^2^ = 41.1, *p* < 0.001). However, due to the degree and variation of missing data, a meaningful comparison of the strength of the relationship of each variable to mortality required the use of imputation.

Mortality increased with increasing LVESVI and decreasing EF in a non-linear fashion. For example, mortality was lowest for LVESVI < 65 cc/m^2^ with minimal increase in risk up to a LVESVI of 65 cc/m^2^. Beyond this, mortality increased linearly with increasing LVESVI. Similarly, there was modest effect on mortality with decreasing EF down to 30%. For EF ≤30%, mortality increased in a linear fashion as EF decreased.

**Fig. 1:**
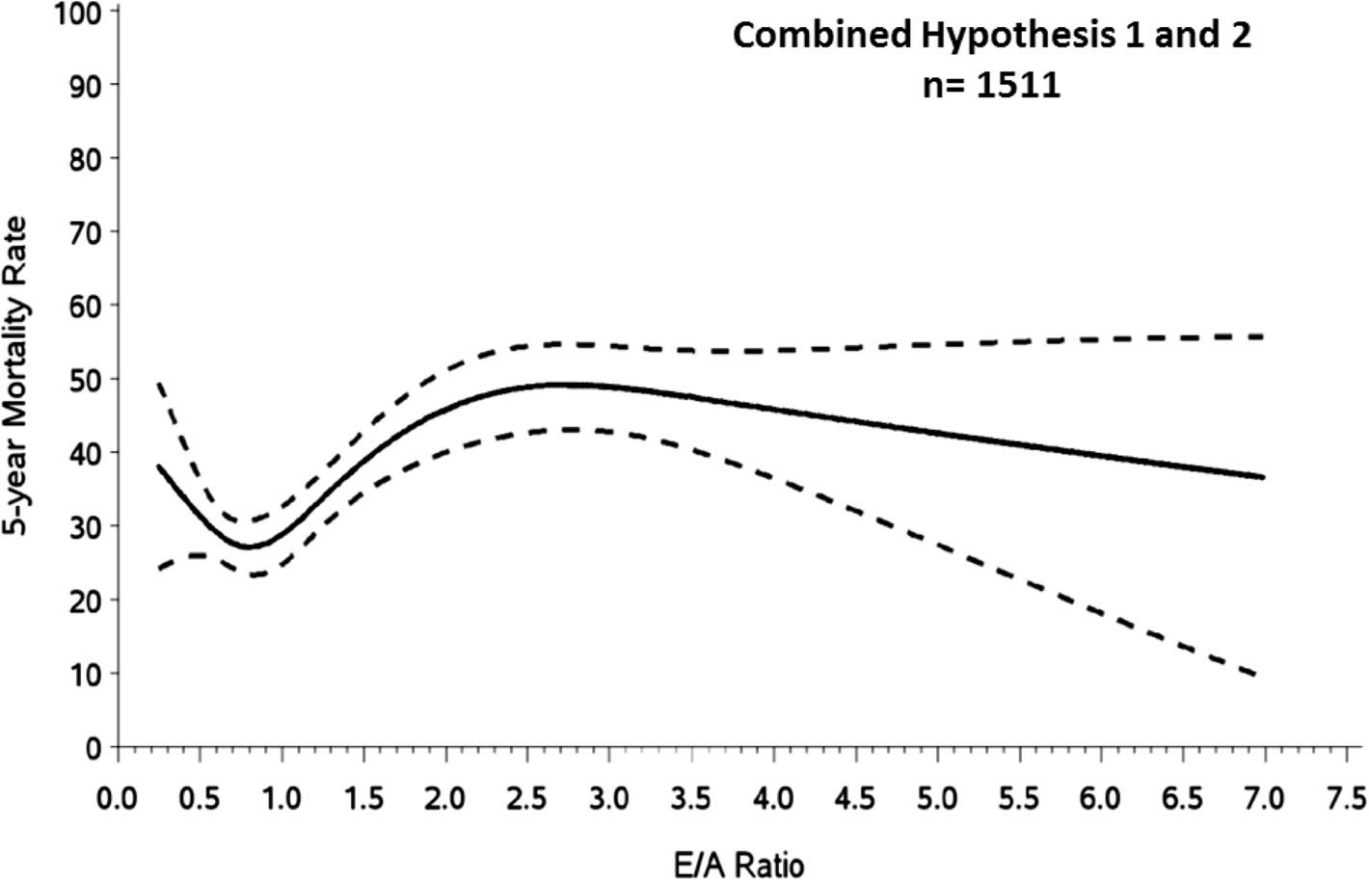
Five year all-cause mortality for the combined cohort (Hypothesis 1 and 2) with increasing E/A ratio in the non-imputed (original) dataset; dotted lines indicate 95% confidence interval. Mortality risk was higher in patients with low (< 0.6) and high (> 1.0) E/A ratio and lowest for those with intermediate E/A ratio (0.6–1.0)

E/A ratio had a u-shaped relationship with mortality (Fig. 1). Mortality was higher for small (E/A < 0.6) and large (> 1.0) values than intermediate (0.6–1.0) values, and was lowest for E/A ≈ 0.8. However, the higher mortality for E/A < 0.6 was not significantly different from mortality with E/A 0.6–1.0 (HR 1.16, 95% CI 0.88–1.53, *p* = 0.28). In contrast, mortality was highest for large E/A ratios (> 1.0 up to approximately 2.3, beyond which there was no further increase in risk) compared to E/A < 0.6 (HR 1.35, 95% CI 1.04–1.75, *p* = 0.02) and E/A 0.6–1.0 (HR 1.58, 95% CI 1.30–1.92, *p* < 0.001).

Of the 1511 patients, 273 had E/A ratio ≤ 0.6, 1010 had E/A > 0.6–2.3 (*n* = 622 with E/A 0.6–1.0), and 228 had E/A > 2.3. Differences in baseline characteristics for each E/A group are shown (Table 2). The E/A ratio > 2.3 group had worse renal function, lower EF, larger LVESVI, more spherical LV, worse RV function, lower systolic blood pressure, higher heart rate and RAR mortality index compared to the other groups. The E/A ≤ 0.6 and E/A > 0.6–2.3 groups were similar, except that those with E/A ≤ 0.6 were older, and more often female. Cardiac index was similar across the three groups.

**Table 2:** Baseline Characteristics by E/A Ratio

	E/A ≤ 0.6	E/A > 0.6–2.3	E/A > 2.3	*P* value
No. Patients	273	1010	228	
Age- yrs. (SD)	63.9 (9.0)	60.3 (9.6)	58.4 (9.2)	< 0.001
Female (%)	50 (18.3)	163 (16.1)	20 (8.8)	0.007
Body mass index-m^2^ (SD)	27.2 (3.9)	27.8 (4.8)	26.7 (4.8)	0.001
Systolic blood pressure-mmHg (SD)	122.3 (18.2)	121.7 (17.3)	115.9 (17.6)	< 0.001
Heart rate-bpm (SD)	71.8 (10.8)	72.0 (13.5)	77.4 (16.1)	< 0.001
Hemoglobin-g/dL (SD)	14.0 (1.60)	13.7 (1.7)	13.4 (1.9)	0.001
Creatinine-mg/dL (SD)	1.14 (0.52)	1.13 (0.56)	1.18 (0.32)	0.001
Blood urea nitrogen-mg/dL (SD)	28.7 (18.5)	27.3 (19.8)	33.2 (23.5)	0.001
EF-% (SD)	30.0 (8.2)	30.1 (8.1)	26.5 (8.11)	< 0.001
LVESVI-cc/m^2^ (SD)	80.0 (30.7)	81.4 (29.8)	92.3 (31.5)	0.001
Cardiac index-L/min/m^2^ (SD)	2.35 (0.70)	2.37 (0.79)	2.42 (0.88)	0.8793
E/e’ (SD)	12.3 (6.2)	17.0 (8.4)	26.9 (12.0)	< 0.001
Deceleration time-ms (SD)	243.5 (52.3)	189.4 (50.3)	135.8 (27.2)	< 0.001
Normal RV function (%)	224 (88.2)	776 (83.6)	96 (43.6)	< 0.001
Sphericity index (SD)	0.67 (0.08)	0.68 (0.09)	0.71 (0.08)	< 0.001
Beta Blocker (%)	236 (86.4)	892 (88.3)	196 (86.0)	0.503
ACE inhibitor (%)	219 (80.2)	826 (81.8)	188 (82.5)	0.787
Nitrates (%)	170 (62.3)	567 (56.1)	106 (46.3)	0.002
Diuretic (%)	170 (62.3)	561 (55.5)	167 (73.6)	< 0.001
Mortality Risk at randomization index	12.9 (8.5)	12.1 (8.9)	14.5 (9.2)	0.001

### Univariable modeling of prognostic variables with imputed datasets

Univariable modeling using the combined 25 imputed datasets are shown (Table 3). LVEDVI was excluded due to its high correlation with LVESVI (*r* = 0.96). E/A ratio was a significant predictor of all-cause mortality (χ^2^ 41.1, *p* < 0.001). Only LVEF (χ^2^ 43.1, *p* < 0.001; HR 0.94, 95% CI 0.93–0.96) and LVESVI (χ^2^ 41.1, *p* < 0.001; HR 1.11, 95% CI 1.07–1.14) were more significant predictors of mortality than E/A ratio.

**Table 3:** Univariable Model for All-Cause Mortality Cased on 25 Imputed Datasets

Echo Variables	Chi Square	DF	*P* value	Hazard Ratio (95% CI)
EF (≤ 30%; Δ1%)	43.1	1	< 0.001	0.94 (0.93, 0.96)
LVESVI (> 65 cc/m^2^; Δ10 cc/m^2^)	41.1	1	< 0.001	1.11 (1.07, 1.14)
E/A ratio	41.1	2	< 0.001	
E/A ≤ 0.6 (Δ 0.1)	4.9	1	0.027	0.81 (0.68, 0.98)
E/A 0.6–2.3 (Δ 0.1)	41.0	1	< 0.001	1.05 (1.03, 1.06)
E/e’ ratio (12–30; Δ1)	39.5	1	< 0.001	1.05 (1.04, 1.07)
MR severity (CFI grade 0, 1, 2, 3–4)	37.6	1	< 0.001	1.36 (1.23, 1.50)
E velocity (0.70–0.85; Δ1.0 m/s)	31.0	1	< 0.001	1.04 (1.02, 1.07)
LA volume index (Δ 10 cc/m^2^)	28.1	1	< 0.001	1.17 (1.10, 1.24)
RV dysfunction (qualitative grade)	27.3	1	< 0.001	1.34 (1.20, 1.49)
Deceleration time (≤190 ms; Δ 10 ms)	26.0	1	< 0.001	0.92 (0.90, 0.95)
Sphericity index (0.66–0.88; Δ 0.1)	6.7	1	0.011	1.28 (1.06, 1.55)

*EF* Ejection fraction, *LVESVI* Left ventricular end systolic volume index, *MR* Mitral regurgitation, *LA* Left atrium, *RV* Right ventricle

### Multivariable modeling of prognostic variables with imputed datasets

Multivariable models in which each echocardiographic variable was considered together with the eight clinical variables are shown in Table 4. Each echocardiographic variable provided incremental prognostic value to the combined set of eight clinical variables. E/A ratio was the third most significant echo variable (χ^2^ = 29.1, *p* < 0.001), following LVESVI (χ^2^ = 44.3, *p* < 0.001) and LVEF (χ^2^ = 31.9, *p* < 0.001).

**Table 4:** Incremental Prognostic Effect of Echo Variables after Adjusting for Clinical Co-variables^a^

	Adjusted Effect of ECHO Variable			Adjusted Hazard Ratio (95% CI)
Models	Wald Chi-Square	DF	***P***-value	
LVESVI (> 65; Δ10 cc/m^2^)	44.3	1	< 0.001	1.12 (1.08, 1.15)
LVEF (≤ 30%; Δ1%)	31.9	1	< 0.001	0.95 (0.93, 0.97)
E/A ratio	29.1	2	< 0.001	
E/A ≤ 0.6 (Δ 0.1)				0.87 (0.72, 1.05)
E/A 0.6–2.3 (Δ 0.1)				1.04 (1.03, 1.06)
MR severity (CFI grade 0, 1, 2, and 3–4)	25.9	1	< 0.001	1.31 (1.18, 1.45)
Deceleration time (≤190 ms, Δ10ms)	23.4	1	< 0.001	0.92 (0.90, 0.95)
E velocity (0.70–0.85; Δ1.0 m/s)	19.4	1	< 0.001	1.03 (1.02, 1.05)
E/e’ ratio (12–30; Δ1)	18.7	1	< 0.001	1.04 (1.02, 1.06)
LA volume index (Δ10 cc/m^2^)	16.2	1	< 0.001	1.14 (1.07, 1.22)
RV dysfunction (by quantitative grade)	16.2	1	< 0.001	1.26 (1.13, 1.41)
Sphericity index (0.66–0.88; Δ 0.1)	10.2	1	0.002	1.35 (1.12, 1.63)

^a^Clinical co-variables: surgical (CABG or CABG+SVR) vs medical treatment, age, creatinine, hemoglobin, history of stroke, history of atrial flutter/fibrillation, diabetes, and NYHA class

*EF* Ejection fraction, *LVESVI* Left ventricular end systolic volume index, *MR* Mitral regurgitation, *LA* Left atrium, *RV* Right ventricle

The combined clinical and echocardiographic multivariable mortality model is shown in Table 5. Creatinine (χ^2^ = 30.0, *p* < 0.001; HR 3.21 95% CI 2.11–4.86), LVESVI (χ^2^ = 27.3, *p* < 0.001; HR 1.09 95% CI 1.06–1.13), age, and E/A ratio (χ^2^ = 12.4, *p* < 0.001) combined accounted for 74% of the prognostic information for predicting risk. Increasing E/A ratio (χ^2^ = 12.4, *p* < 0.001) was the 5th most important variable after age and treatment strategy (MED vs CABG or CABG +SVR).

**Table 5:** Multivariable Mortality Model

Variables	Wald Chi Square	DF	*P* value	Hazard Ratio (95% CI)
Creatinine (1.0–1.6; Δ1mg/dL)	30.0	1	< 0.001	3.21 (2.11, 4.86)
LVESVI (> 65; Δ10 cc/m^2^)	27.3	1	< 0.001	1.09 (1.06, 1.33)
Age (> 57; Δ10 yr)	20.3	1	< 0.001	1.35 (1.19, 1.55)
Treatment (CABG/ CABG +SVR vs. MED)	13.1	1	< 0.001	0.71 (0.59, 0.85)
E/A ratio	12.4	2	< 0.001	
E/A ≤ 0.6 (Δ0.1)	1.4	1	0.237	0.89 (0.73, 1.08)
E/A 0.6–2.3 (Δ0.1)	12.4	1	< 0.001	1.03 (1.01, 1.04)
NYHA (I, II, III, IV)	7.3	1	0.007	1.18 (1.05, 1.34)
Diabetes (yes or no)	6.2	1	0.013	1.27 (1.05, 1.54)
History of Stroke (yes or no)	6.2	1	0.013	1.49 (1.09, 2.04)
MR severity (CFI grade)	6.1	1	0.014	1.15 (1.03, 1.29)
History of atrial fibrillation/flutter (yes or no)	4.2	1	0.041	1.34 (1.01, 1.78)
Hemoglobin (≤14.3Δ 1 g/dL)	3.2	1	0.076	0.94 (0.88, 1.01)

*EF* Ejection fraction, *LVESVI* Left ventricular end systolic volume index, *MR* Mitral regurgitation, *LA* Left atrium, *RV* Right ventricle, *CABG* Coronary artery bypass grafting, *SVR* Surgical ventricular reconstruction

### Interaction with treatment strategy

#### Hypothesis 1 (MED vs. CABG)

**Fig. 2:**
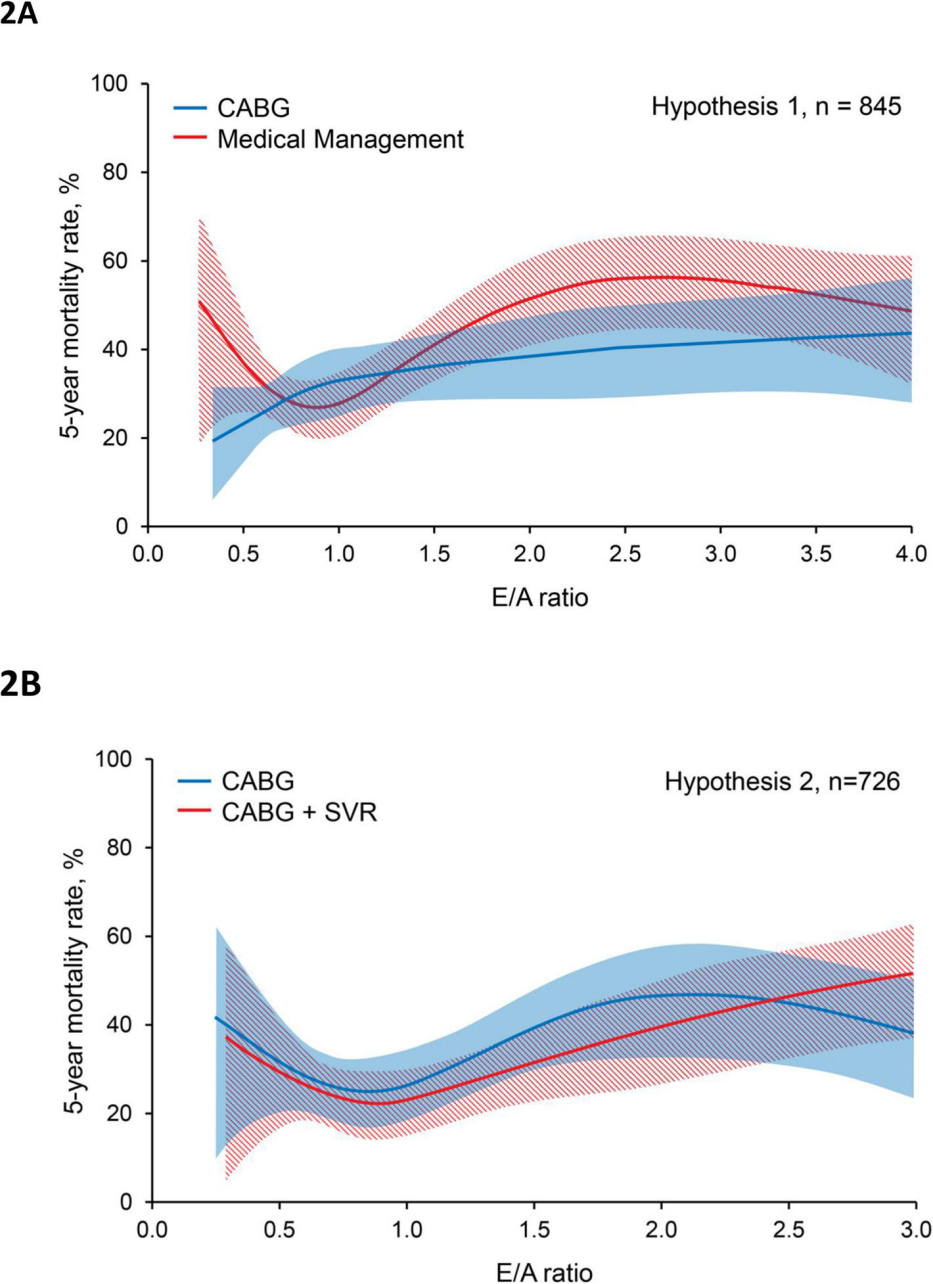
Five-year all-cause mortality by treatment. **a** Hypothesis 1: CABG vs. Medical Therapy. Medical treatment arm is shown in red; shaded area indicates 95% confidence interval. CABG arm is shown in blue; shaded area indicates 95% confidence interval. **b** Hypothesis 2: CABG vs. CABG + SVR. CABG + SVR arm is shown in red; shaded area indicates 95% confidence interval. CABG arm is shown in blue; shaded area indicates 95% confidence interval

Of the 1511 patients included in this analysis, 845 were enrolled in H1 (60 were enrolled in both H1 and H2) [9, 23]. Only E/A ratio had a significant treatment interaction (*p* = 0.033). While the medical treatment arm displayed a u-shaped relationship with mortality (Fig. 2a), in the CABG arm, mortality increased in a more linear fashion as E/A increased. Compared to the medical arm, mortality was lower for CABG with smaller E/A ratio ≤ 0.6 (HR 0.64 95% CI 0.36, 1.14, *p* = 0.13) and higher E/A ratio > 1.4 (HR 0.78, 95% CI 0.54, 1.12, *p* = 0.17), although this difference was not statistically significant. Mortality risk was similar for CABG and medical therapy when E/A ratio approached 0.8 (HR 1.05, 95% CI 0.75, 1.47, *p* = 0.79).

#### Hypothesis 2 (CABG vs. CABG +SVR)

The remaining 726 patients in our study cohort had sufficient anterior and apical dyskinesia to be eligible for SVR and were enrolled in H2 [9, 11]. In H2, only RV dysfunction (*p* = 0.038) had a significant treatment interaction. With normal RV function (680 patients), there was no difference in mortality with CABG versus CABG +SVR. With mild RV dysfunction (100 patients), there was a trend towards increased mortality with CABG+SVR versus CABG alone (HR 1.47 95% CI 0.71–3.04, *p* = 0.301). However, with moderate or severe RV dysfunction (76 patients), mortality was significantly higher with CABG +SVR compared to CABG alone (HR 3.05 95% CI 1.29–7.22, *p* = 0.008). LVESVI, EF, MR severity, and E/A ratio did not have a significant treatment interaction in H2. E/A ratio exhibited a u-shaped relationship with mortality for CABG and CABG + SVR without any significant difference in mortality between the two groups (Fig. 2b).

## Discussion

We observed that baseline echocardiographic markers of LV remodeling can contribute as incremental prognostic values to current clinical risk markers in ischemic HFrEF. Diastolic dysfunction was a stronger predictor of mortality than NYHA functional class, anemia, diabetes, history of atrial fibrillation and stroke. When combined with advancing renal dysfunction and elderly age, larger LVESVI and worsening diastolic dysfunction accounted for 74% of the prognostic information in predicting risk of death. Mortality of those with less advanced diastolic dysfunction may be lower when treated with surgical revascularization compared to medical therapy. We did not observe a treatment interaction with E/A ratio for CABG vs. CABG + SVR.

The degree of LV remodeling in HFrEF is variable and reflects differences in hemodynamics, cardiac structure, and geometry [3, 5, 6, 18, 24]. For example, while the STICH cohort all had EF ≤ 35%, diastolic function varied widely, with mild diastolic dysfunction in one-third and severe diastolic dysfunction in only one-fifth [3]. RV function was normal in most of the cohort (75.5% of 1838 patients with baseline RV function assessment) but < 5% had severe RV dysfunction [3]. MR severity and LV volumes also varied [3]. Progression of RV and diastolic dysfunction, functional MR, and LV enlargement suggests more advanced cardiac remodeling. Consistent with previous studies [5, 6], we observed that each of these was associated with increased mortality risk and could explain differences in mortality amongst HFrEF patients with similar EF. Decreased EF was associated with mortality risk with univariable modeling but not when other echo variables were included in multivariable analysis, supporting the notion that these markers of LV remodeling provide prognostic information beyond EF alone.

Amongst markers of advanced LV remodeling, diastolic dysfunction may be more important prognostic marker than abnormal LV geometry. We previously demonstrated that increased LVESVI and sphericity index influences poor survival following CABG or CABG+SVR [4, 25], but in our current analysis, diastolic function outperformed sphericity index. Whereas previous HFrEF studies have identified deceleration time, E/e’ ratio or diastolic function grade as prognostic diastolic function variables [5, 6], we found that E/A ratio was the most robust indicator in HFrEF due to ischemic etiology. E/A ratio is the most easily obtainable diastolic function parameter and is recommended by the American Society of Echocardiography and European Association of Cardiovascular Imaging joint guidelines as the first parameter to grade diastolic function and estimate LV filling pressure [15]. Since it reflects transmitral gradient and captures dynamic changes in LV filling pressure, E/A can discriminate HFrEF with optimized hemodynamics from decompensated HF. In contrast, the mitral annulus early diastolic velocity (e’) reflects the status of myocardial relaxation [17], and is reduced in almost all HFrEF patients, resulting in increased E/e’ ratio. Therefore, E/A ratio is a better prognostic parameter than E/e’ ratio.

While our data do not replace the diastolic function grading recommended by the ASE and EACVI [15], they do have implications for the optimal diastolic filling pattern in HFrEF due to ischemic etiology. We found that mortality risk was higher in patients with low (< 0.6) and high (> 1.0) E/A ratio and lowest for those with intermediate E/A ratio (0.6–1.0). Similar findings were observed in the Strong Heart Study, a population based cohort of middle aged to elderly Native Americans [26]. While higher mortality is expected in HFrEF patients with E/A > 1.0 suggesting advanced diastolic dysfunction [6], an impaired relaxation pattern (E/A < 0.6) is usually considered to be an optimal diastolic filling pattern. One explanation could be that poor outcomes with E/A < 0.6 reflected hemodynamic compromise, but cardiac output and systolic blood pressure were similar to patients with E/A 0.6–2.3. Another possibility is that intravascular volume depletion due to diuresis or other mechanisms contributes to lower than ideal preload in those with E/A ratio < 0.6.

E/A ratio ≥ 2.0 is a restrictive diastolic filling pattern usually portending the worst prognosis [6], but surprisingly, we observed that E/A ratio > 2.3 was associated with minimal further increased mortality risk. However, this group also had lower systolic blood pressure, hemoglobin, and EF, larger LVESVI, and worse renal function, and perhaps when E/A ratio is severely elevated these factors had a greater effect on mortality.

Restrictive diastolic filling has been shown to be associated with reduced myocardial viability and poor survival after CABG [27]. We also observed increasing mortality with CABG as E/A ratio increased up to 2.3, but mortality trended higher in this group in the medical treatment arm compared to CABG, with similar trends for E/A ratio < 0.6. In contrast, mortality in both treatment groups was lowest when E/A closest 0.8, further emphasizing that this is the optimal filling pattern in ischemic HFrEF and that treatment, such as diuresis, should be tailored to avoid E/A < 0.6 or > 1.0. Whether those with E/A < 0.6 or > 1.0 will have better survival with medical therapy or CABG is less certain, as the differences in mortality were not statistically significant.

We did not find treatment interactions with E/A ratio in H2 (CABG versus CABG + SVR), although moderate to severe RV dysfunction predicted worse survival with SVR, consistent with our recent report [12]. In H2, RV dysfunction was also associated with increased E/A ratio, advanced HF, and lower cardiac index [12], suggesting more advanced LV remodeling. We also previously demonstrated that smaller LVESVI and more preserved EF, both markers of less advanced LV remodeling, could identify a subgroup of STICH patients more likely to benefit from SVR [4]. Together, these data suggest that echocardiographic variables could identify patients with less advanced remodeling more likely to benefit from SVR that could not be determined by risk stratification based on clinical characteristics or EF alone [22].

All echocardiographic variables in our model conferred incremental mortality risk to clinical data but the combination of increased creatinine, elderly age, larger LVESVI, and abnormal E/A ratio, had the most influence on all-cause mortality. At present, the most commonly applied HF prognostic models, such as the Seattle Heart Failure Model and the Heart Failure Survival Score, synthesize clinical data and EF to estimate prognosis but do not include other echocardiographic markers [7, 8], and models that have proposed the addition of echocardiographic markers are not widely applied [28]. Our data are not only consistent with previous studies demonstrating the prognostic importance of advanced diastolic dysfunction and LV enlargement, but show that these are more powerful predictors than many other clinical markers. Our data argue for inclusion of diastolic dysfunction and LV enlargement markers in future prognostic models and decision aids to determine optimal treatment strategy in ischemic HFrEF.

### Limitations

Our analysis has limitations. First, excluding 498 patients without measureable E/A ratio may have introduced bias by excluding 118 patients with > moderate MR. Second, in the small number (7.8%; 111 of 1511) of patients with > moderate MR included, E/A ratio could reflect the severity of MR rather than diastolic function. Third, the results presented are based on LV diastolic function subgroup analyses of the overall study population. The perils of subgroup analysis are well documented, and thus cautious interpretation is required. Fourth, multiple imputation was required to create complete datasets due to additional missing echocardiographic data. However, this technique is a well-established method which permitted inclusion of all 1511 patients with E/A ratio and strengthened our analysis. Fifth, the sphericity index is an important index in decisions for a role in the for surgical ventricular reconstruction. However this was evaluated in 935 patients who made up only 62% of our study population.. Sixth, RV systolic pressure was not included in the model because of a large number (> 80%) of missing data. Finally, our analysis of clinical risk markers for HF was limited to data prospectively collected during the STICH trial and did not include functional studies (peak VO_2_) or biomarkers.

## Conclusions

Diastolic dysfunction and echocardiographic markers of advanced LV remodeling can contribute incremental prognostic value to current clinical markers of HF severity. Inclusion of E/A ratio and LVESVI could enhance prognostic models for ischemic HF and influence treatment strategy. E/A ratio closest to 0.8 is the most optimal filling pattern in ischemic HFrEF and treatments which affect preload, such as diuresis, should be adjusted to maintain E/A > 0.6 and < 1.0.

## Data Availability

The datasets used and/or analysed during the current study are available from the corresponding author on reasonable request.

## Abbreviations

STICH: Surgical Treatment for Ischemic Heart Failure
LV: Left ventricle
HFrEF: Heart failure with reduced ejection fraction
RV: Right ventricle
MR: Mitral regurgitation
LVEDVI: Left ventricular end-diastolic volume index
LVESVI: Left ventricular end-systolic volume index
CABG: Coronary artery bypass grafting
SVR: Surgical ventricular revascularization
RAR: Risk randomization index

## References

[1] Lin G, Gersh BJ, Greene EL, Redfield MM, Hayes DL, Brady PA (2011). Renal function and mortality following cardiac resynchronization therapy. Eur Heart J.

[2] Al-Ahmad A, Rand WM, Manjunath G, Konstam MA, Salem DN, Levey AS (2001). Reduced kidney function and anemia as risk factors for mortality in patients with left ventricular dysfunction. J Am Coll Cardiol.

[3] Oh JK, Pellikka PA, Panza JA, Biernat J, Attisano T, Manahan BG (2012). Core lab analysis of baseline echocardiographic studies in the STICH trial and recommendation for use of echocardiography in future clinical trials. J Am Soc Echocardiogr.

[4] Oh JK, Velazquez EJ, Menicanti L, Pohost GM, Bonow RO, Lin G (2013). Influence of baseline left ventricular function on the clinical outcome of surgical ventricular reconstruction in patients with ischaemic cardiomyopathy. Eur Heart J.

[5] Grayburn PA, Appleton CP, DeMaria AN, Greenberg B, Lowes B, Oh J (2005). Echocardiographic predictors of morbidity and mortality in patients with advanced heart failure: the Beta-blocker evaluation of survival trial (BEST). J Am Coll Cardiol.

[6] Pinamonti B, Zecchin M, Di Lenarda A, Gregori D, Sinagra G, Camerini F (1997). Persistence of restrictive left ventricular filling pattern in dilated cardiomyopathy: an ominous prognostic sign. J Am Coll Cardiol.

[7] Levy WC, Mozaffarian D, Linker DT, Sutradhar SC, Anker SD, Cropp AB (2006). The Seattle heart failure model: prediction of survival in heart failure. Circulation..

[8] Aaronson KD, Schwartz JS, Chen T-M, Wong K-L, Goin JE, Mancini DM (1997). Development and prospective validation of a clinical index to predict survival in ambulatory patients referred for cardiac transplant evaluation. Circulation..

[9] Velazquez EJ, Lee KL, O’Connor CM, Oh JK, Bonow RO, Pohost GM (2007). The rationale and design of the surgical treatment for ischemic heart failure (STICH) trial. J Thorac Cardiovasc Surg.

[10] Jones RH, White H, Velazquez EJ, Shaw LK, Pietrobon R, Panza JA (2010). STICH (surgical treatment for ischemic heart failure) trial enrollment. J Am Coll Cardiol.

[11] Jones RH, Velazquez EJ, Michler RE, Sopko G, Oh JK, O'Connor CM (2009). Coronary bypass surgery with or without surgical ventricular reconstruction. N Engl J Med.

[12] Kukulski T, She L, Racine N, Gradinac S, Panza JA, Velazquez EJ (2015). Implication of right ventricular dysfunction on long-term outcome in patients with ischemic cardiomyopathy undergoing coronary artery bypass grafting with or without surgical ventricular reconstruction. J Thorac Cardiovasc Surg.

[13] Anavekar NS, Gerson D, Skali H, Kwong RY, Kent Yucel E, Solomon SD (2007). Two-dimensional assessment of right ventricular function: an echocardiographic–MRI correlative study. Echocardiography..

[14] Lang RM, Bierig M, Devereux RB, Flachskampf FA, Foster E, Pellikka PA (2005). Recommendations for chamber quantification: a report from the American Society of Echocardiography's guidelines and standards committee and the chamber quantification writing group, developed in conjunction with the European Association of Echocardiography, a branch of the European Society of Cardiology. J Am Soc Echocardiogr.

[15] Nagueh SF, Smiseth OA, Appleton CP, Byrd BF, Dokainish H, Edvardsen T (2016). Recommendations for the evaluation of left ventricular diastolic function by echocardiography: an update from the American Society of Echocardiography and the European Association of Cardiovascular Imaging. Eur J Echocardiogr.

[16] Di Donato M, Castelvecchio S, Brankovic J, Santambrogio C, Montericcio V, Menicanti L (2007). Effectiveness of surgical ventricular restoration in patients with dilated ischemic cardiomyopathy and unrepaired mild mitral regurgitation. J Thorac Cardiovasc Surg.

[17] Ommen SR, Nishimura R, Appleton CP, Miller FA, Oh JK, Redfield MM (2000). Clinical utility of Doppler echocardiography and tissue Doppler imaging in the estimation of left ventricular filling pressures: a comparative simultaneous Doppler- catheterization study. Circulation..

[18] White HD, Norris RM, Brown MA, Brandt PW, Whitlock RM, Wild CJ (1987). Left ventricular end-systolic volume as the major determinant of survival after recovery from myocardial infarction. Circulation..

[19] Cox DR (1972). Regression models and life-tables. J Royal Statist Soc B.

[20] Harrell FE (2001). Regression modeling strategies: with applicaitons to linear models, logistic regression, and survival analysis.

[21] Rubin DB (2004). Multiple imputation for nonresponse in surveys: John Wiley & Sons.

[22] Zembala M, Michler RE, Rynkiewicz A, Huynh T, She L, Lubiszewska B (2010). Clinical characteristics of patients undergoing surgical ventricular reconstruction by choice and by randomization. J Am Coll Cardiol.

[23] Velazquez EJ, Lee KL, Deja MA, Jain A, Sopko G, Marchenko A (2011). Coronary-artery bypass surgery in patients with left ventricular dysfunction. N Engl J Med.

[24] St John Sutton M, Pfeffer MA, Plappert T, Rouleau JL, Moyé LA, Dagenais GR (1994). Quantitative two-dimensional echocardiographic measurements are major predictors of adverse cardiovascular events after acute myocardial infarction. The protective effects of captopril. Circulation..

[25] Choi J-O, Daly RC, Lin G, Lahr BD, Wiste HJ, Beaver TM (2015). Impact of surgical ventricular reconstruction on sphericity index in patients with ischaemic cardiomyopathy: follow-up from the STICH trial. Eur J Heart Fail.

[26] Bella JN, Palmieri V, Roman MJ, Liu JE, Welty TK, Lee ET (2002). Mitral ratio of peak early to late diastolic filling velocity as a predictor of mortality in middle-aged and elderly adults: the strong heart study. Circulation..

[27] Yong Y, Nagueh SF, Shimoni S, Shan K, He Z-X, Reardon MJ (2001). Deceleration time in ischemic cardiomyopathy: relation to echocardiographic and Scintigraphic indices of myocardial viability and functional recovery after revascularization. Circulation..

[28] Senni M, Parrella P, De Maria R, Cottini C, Böhm M, Ponikowski P (2013). Predicting heart failure outcome from cardiac and comorbid conditions: the 3C-HF score. Int J Cardiol.

